# Influences of the Common *FTO* rs9939609 Variant on Inflammatory Markers Throughout a Broad Range of Body Mass Index

**DOI:** 10.1371/journal.pone.0015958

**Published:** 2011-01-05

**Authors:** Esther Zimmermann, Kristin Skogstrand, David M. Hougaard, Arne Astrup, Torben Hansen, Oluf Pedersen, Thorkild I. A. Sørensen, Tine Jess

**Affiliations:** 1 Institute of Preventive Medicine, Copenhagen University Hospital, Copenhagen, Denmark; 2 Institute of Biomedical Sciences, University of Copenhagen, Copenhagen, Denmark; 3 Department of Clinical Biochemistry and Immunology, Statens Serum Institut, Copenhagen, Denmark; 4 Department of Human Nutrition, Faculty of Life Sciences, University of Copenhagen, Copenhagen, Denmark; 5 Hagedorn Research Institute, Gentofte, Denmark; 6 Faculty of Health Science, University of Aarhus, Aarhus, Denmark; Brigham and Women's Hospital/Harvard Medical School, United States of America

## Abstract

**Background:**

A recent study reported that the fatness associated A-allele of *FTO* rs9939609 increased plasma high sensitivity C-reactive protein (hs-CRP) levels independent of fatness. We aimed to investigate if this gene variant had fatness-independent effects on plasma hs-CRP and 10 additional circulating obesity-related adipokines throughout a broad range of body mass index (BMI) among Danish men.

**Methodology/Principal Findings:**

In a population of 362,200 young men, examined for military service between 1943 and 1977, two groups were identified: 1) a random 1% sample and 2) all obese men (BMI = 31.0 kg/m^2^, all of whom were above the 99^th^ percentile of this population). At an average age of 49 years (range: 39 through 65 years), 551 men, hereof 231 of the obese, were re-examined, including genotyping and measurement of the fasting circulating inflammatory markers hs-CRP, IL-1β, IL-6, IL-10, IL-18, mip1α, mip1β, sTNFα-R1, TGF-β, TNF-α and leptin. Men with known disease were excluded from the examination. All the inflammatory markers were log-transformed to approximate a normal distribution. Genotype-phenotype relationships were studied using linear regression analyses with the inflammatory markers as the response variable. Significant positive associations between hs-CRP, leptin and a broad range of BMI were observed, but the associations did not significantly differ across *FTO* rs9939609 genotype. There were no significant associations between the other inflammatory markers, *FTO* rs9939609 genotype or BMI, respectively.

**Conclusion:**

No fatness-independent effects of the *FTO* rs9939609 A-allele on a series of inflammatory markers were observed in this cohort of healthy middle-aged men representing a broad range of fatness.

## Introduction

Along with the rising prevalence of obesity worldwide [1], [2], an increasing focus on the role of obesity-related inflammation has evolved, primarily confined to its role in development of metabolic complications to obesity such as type 2 diabetes and cardiovascular disease [3]. Plasma C-reactive protein (CRP) is the obesity-related inflammatory marker that has been most consistently associated with cardiovascular risk [4], [5]. However, a range of other systemically measurable inflammatory markers have been suggested to associate not only with obesity [6], but putatively also with morbidity and mortality [4], [7], [8], and these markers are therefore also of clinical interest.

There is an obvious lack in the understanding of causes behind and modifiers of this obesity-related inflammatory activity and environmental as well as genetic factors may be considered to play a role in the aetiology of this condition. Hitherto, the A-allele of the *FTO* rs9939609 is the genetic variant most strongly associated with common obesity [9]–[11]. A recent study including 2,415 participants from a middle-aged German population reported that the *FTO* rs9939609 A-allele causes variation in CRP levels independent of its effect on fatness [12].

In addition to CRP, it is thus of interest to investigate whether the *FTO* rs9939609 is associated with a range of other inflammatory markers. The aim of the present study was to investigate whether the *FTO* rs9939609 A-allele has fatness-independent effects on circulating levels of the inflammatory markers hs-CRP, interleukin (IL)-1β, IL-6, IL-10, IL-18, tumor necrosis factor-alfa (TNF-α), soluble tumour necrosis factor α receptor antagonist (sTNFα-R1), transforming growth factor-beta (TGF-β), macrophage inflammatory proteins (MiP)-1α, and MiP-1β. Several of these markers are secreted from the adipose tissue, so-called adipokines, which also includes the non-inflammatory marker leptin. Leptin was included in the analyses as an adipocyte marker.

## Methods

The participants included in this study were originally identified among a total of 362,200 Danish men who underwent the mandatory draft board examination in Copenhagen during 1943–1977 and in the remainder of Sjælland during 1964–1977 [13]–[15]. Among these, a study population consisting of a randomly selected 1.0% sample (n = 3,601) and all men with a body mass index (BMI) equal to or above 31.0 kg/m^2^ (n = 1,930) was sampled manually from the draft board files during the 1970s. The latter group represented all at least 35% overweight men according to a national standard in use at the time of sampling (all of whom were above the 99^th^ percentile of the BMI distribution). This sampling design provided coverage of the entire distribution of BMI in the population with a 100-fold relative over-sampling of the obese, in order to allow particular focus on obesity and related aspects. All obese and half of the controls were invited to participate in the examination programme of the Copenhagen City Heart Study in 1981–83 and again in 1991–93. Details of these surveys are published elsewhere [16], [17].

A last thorough whole-day examination was conducted in 1998–2000 among subjects who participated in the Copenhagen City Heart Study in 1991–93. Exclusion criteria were age above 65 years (to avoid bias by aging), residence too far away from Copenhagen to allow one-day examination, refusal in the past to participate in follow-up examinations, regular medication and known disease. Details of this survey is published elsewhere [11], [18]. In total, 551 men, hereof 231 of the obese were re-examined, including genotyping and measurement of fasting circulating inflammatory markers at an average age of 49 years (range: 39 through 65 years).

### Genotyping

Genotyping of *FTO* rs9939609 (Taqman allelic discrimination; KBiosciences, Cambridge, UK) was successful in 97% of the samples with a genotype error rate of 0.27% based on 1,464 duplicate samples. All genotype groups obeyed the Hardy–Weinberg equilibrium and the A-allele frequency was 0.41 in controls and 0.51 in obese individuals. Molecular genetic analysis, including genotyping of the *FTO* SNP rs9939609, was conducted on 551 men, hereof 231 of the originally obese. At present, the SNPs that are strongest associated with BMI belong to a linkage disequilibrium (LD) block encompassing parts of the first two introns of *FTO* [19]. Since the SNPs are in tight LD, the study of one of them will convey the effect.

### Phenotypic and biochemical measurements

Objective measures of height and weight were obtained [20]. BMI was calculated as weight per height squared. Furthermore, fat mass was assessed by dual-energy x-ray absorptiometry (DXA-IQ DEXA; Lunar, Madison, WI). Fat-BMI is equivalent with BMI, but calculated as weight of body fat per height squared. Hip and waist circumference were measured to the nearest 0.5 cm with the subjects standing. Hip circumference was measured at the maximal width over the greater trochanters, and waist circumference was measured midway between the iliac crest and the rib cage. Blood samples were obtained in the morning after an overnight 12-hour fast, and hs-CRP was measured in plasma by high sensitivity enzyme-linked immunosorbent assay (ELISA) [21], with a lower detection limit of 0.005 ìg/dL. The concentration of the cytokines in serum, IL-1β, IL-6, IL-10, IL-18, TNF-α, sTNFα-R1, TGF-β, MiP-1α, and MiP-1β, were measured using the Luminex® xMAP technology as described in Skogstrand et al. [22]. Serum leptin was assessed by RIA (human leptin RIA kit, Alta Diagnostica, Marburg, Germany). The serum levels were measured in the same lab and in the same batches for all samples. The lower and upper detectable concentration limits, as well as the number of measures within these limits, can be seen in Table S2.

### Statistical analysis

The inflammatory markers were modelled as the response variable in both the linear and the logistic regression analyses. In the linear regressions only detectable measurements were included in the analyses. The distribution of the inflammatory markers were visually tested via Q-Q plots, and as they all departed from a normal distribution (Shapira-Wilk test, all p<0.0001), they were log(e)-transformed [23]. For the logistic regression analyses the measures either above or below the detection limits of the assays were recoded to correspond to these limits, and then all the measurements of the markers, whether within or beyond the detection limits, were dichotomized by the median (the number of measures above or below these limits can be seen in Table S2). BMI was also log-transformed (base 1.1) to pull in the long right tail, and hereby obtain a better model fit in regard to the underlying assumptions of linearity in both the linear and logistic regression models [23]. Waist (for given BMI) was modelled per the 10 cm increase, and waist-to-hip ratio (WHR) was log-transformed (base 1.1). Both the likelihood ratio test and the Akaike information criterion (AIC) showed that an additive model of *FTO* rs9939609 gave the best model fit in both the linear and logistic regression models as compared to dominant and recessive models.

Due to the skewed inflammatory markers their characteristics are presented as geometric means (which is the antilog of the arithmetic mean of the logged data) with 95% confidence intervals (CIs). After checking for linearity, all analyses were run for the obese and randomly selected men combined, which was possible because BMI was used only as an independent covariate. We investigated if hs-CRP and the 10 other adipokines were associated with the *FTO* rs9939609 and with the anthropometric obesity measures in our cohort, both in crude analyses, and when mutually adjusted. Then we investigated if an association between BMI and the inflammatory markers differed across *FTO* rs9939609 genotype. The results from the linear regressions are presented as effect estimates with 95% CI, which represent a factor of change in the inflammatory marker relative to the measurement unit in the predicting covariate (i.e. an estimate of 1.17 for *FTO* rs9939609 genotype in relation to hs-CRP is to be interpreted as a 17% higher hs-CRP-level per additional A-allele). The results for the logistic regressions are presented as odds ratios (OR) with 95% CI. Interactions between BMI and *FTO* rs9939609, both modeled as continuous variables (0, 1 and 2 for the *FTO* alleles), were investigated. The likelihood ratio test assessed whether the model with the product term provided a better fit than the model without the product term. All models were adjusted for age. All analyses were two-tailed and a significance level was accepted at p<0.05. Analyses were carried out with Stata (version 9.2; Stata Corporation, College Station, Texas).

### Ethics statement

The Danish Data Protection Agency and the Ethical Committees of Copenhagen and Frederiksberg municipalities approved the study, which was in accordance with the Helsinki Declaration II. All participants signed written consent before participating.

## Results

In Table 1 the phenotype characteristics of the two cohorts of men are presented according to the *FTO* genotype. Men belonging to the obese cohort were obviously heavier, had larger waist circumference and WHR than the randomly selected men. The obese were also younger, which reflects the increasing prevalence of obesity in more recent time [15]. From Table 1 we calculated the OR of having a BMI = 31.0 kg/m^2^ at the draft board examination. Using the TT genotype as the reference group we found that the OR for the TA genotype was 1.29 (95% CI: 0.87–1.93) and for the AA genotype 2.22 (95% CI: 1.38–3.57).

**Table 1:** Characteristics according to *FTO* rs9939609 given as mean (S.D.) of the two study cohorts representing, respectively, a random sample of Danish male draftees and all obese male draftees.

		*FTO* rs9939609			
	Cohort	TT	TA	AA	P*
No of subjects	Random sample	113	146	55	0.001†
	Obese sample	61	102	66	
Age (yrs)	Random sample	49.5 (6.1)	50.2 (5.9)	50.1 (6.6)	0.52
	Obese sample	46.9 (5.0)	48.4 (5.3)	46.7 (4.9)	0.74
BMI (kg/m^2^)	Random sample	25.8 (3.7)	26.1 (3.4)	26.3 (4.5)	0.49
	Obese sample	36.1 (6.4)	35.5 (5.6)	36.4 (5.7)	0.76
Waist circumference (cm)	Random sample	93.3 (11.3)	93.9 (10.2)	94.5 (11.4)	0.48
	Obese sample	117.8 (15.6)	117.8 (14.0)	118.9 (13.3)	0.68
Waist-to-hip ratio	Random sample	0.952 (0.063)	0.957 (0.062)	0.961 (0.064)	0.38
	Obese sample	1.046 (0.073)	1.034 (0.063)	1.037 (0.071)	0.48

Abbreviations: BMI: Body mass index.

*Test for trend.

† Chi^2^-test of the obese vs. randomly sampled according to genotype distribution of *FTO* rs9939609.

The circulating levels of hs-CRP and the adipokines are presented as geometric means in Table 2 for the two cohorts combined (and for the two cohorts separately in Table S1). There was no overall pattern in the distribution of these across *FTO* genotype. For the adipokines IL-1β, IL-10, IL-18, mip1α, mip1β, sTNFα-R1, TGF-β, and TNF- α there were no association with either *FTO* rs9939609 or BMI (analysis not shown, see Table S1). The same results were obtained by the logistic regression analyses of the dichotomized values of the inflammatory markers (Table S2).

**Table 2:** Geometric mean (95% confidence intervals) of the inflammatory markers according to *FTO* rs9939609 genotype for the two cohorts combined.

Inflammatory marker	N	TT		TA		AA		P*
		Mean	95% CI	Mean	95% CI	Mean	95% CI	
hs-CRP (mg/l)	529	1.08	0.88; 1.33	1.27	1.08; 1.50	1.47	1.15; 1.88	0.05
IL-1β (pg/ml)	104	16.41	12.98; 20.76	16.08	12.12; 21.33	12.64	10.61; 15-05	0.35
IL-6 (pg/ml)	377	25.88	22.84; 29.32	26.09	23.38; 29.13	21.32	18.71; 24.29	0.06
IL-10 (pg/ml)	145	21.46	16.42; 28.04	22.96	18.44; 28.58	20.19	15.07; 27.06	0.84
IL-18 (pg/ml)	519	343.75	307.1; 384.70	356.00	323.43; 391.84	373.04	327.53; 424.88	0.36
TNF-α (pg/ml)	197	16.80	13.85; 20.38	17.14	14.14; 20.78	13.97	12.21; 15.97	0.30
STNFα-R1 (pg/ml)	446	578.67	505.46; 662.49	626.68	558.86; 702.73	567.24	495.87; 648.89	0.95
TGF-β (pg/ml)	371	143.75	130.88; 157.88	150.90	141.24; 161.22	143.40	133.86; 153.62	0.95
MiP-1α (pg/ml)	350	42.89	38.10; 48.29	39.33	35.73; 43.30	46.82	38.03; 57.64	0.56
MiP1β (pg/ml)	537	139.09	126.02; 153.50	142.25	131.28; 154.14	143.12	128.04; 159.98	0.69
Leptin (ng/ml)	548	5.51	4.87; 6.24	5.69	5.15; 6.28	6.81	5.89; 7.87	0.04

*Test for trend.

The geometric means of the inflammatory markers according to *FTO* genotype are presented for the two cohorts separately in Table S1.

There was a trend across *FTO* genotype in relation to hs-CRP, IL-6 and leptin (Table 2); hence, these three adipokines were investigated in further detail.

Positive correlations between hs-CRP and fat mass (kg) were observed both among the randomly sampled and the obese men (Spearman's ρ equal to 0.29 (p<0.001) and 0.31 (p<0.001), respectively). Similarly, positive correlation between leptin and fat mass (kg) was observed (Spearman's ρ equal to 0.87 (p<0.001) in the randomly sampled and 0.86 (p<0.001) in the obese). There were no correlations between IL-6 and fat mass (kg) (Spearman's ρ equal to 0.02 (p = 0.81 in the randomly sampled and 0.03 (p = 0.70) in the obese)).

**Figure 1:**
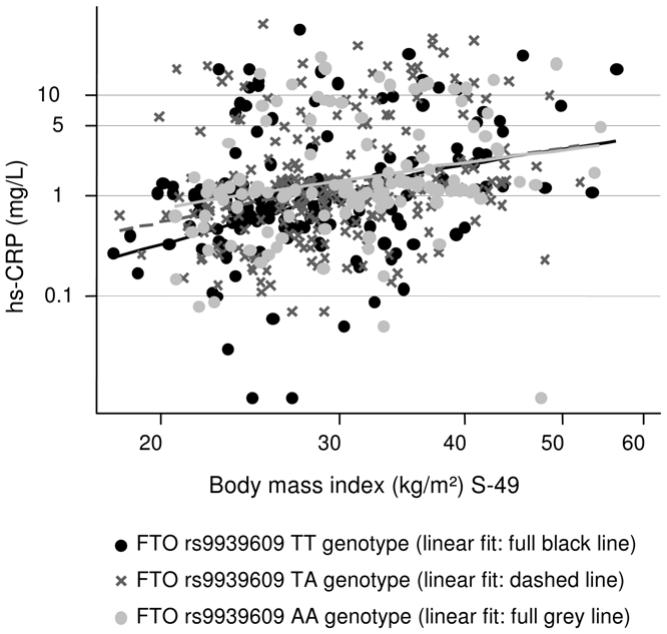
The association between BMI and hs-CRP according to *FTO* rs9939609 genotype. *FTO* rs9939609 genotype had no main effect on hs-CRP when BMI was taken into account (see Table 3); further, this figure shows there was no interaction between BMI and *FTO* rs9939609 genotype in relation to hs-CRP (p = 0.62).

**Figure 2:**
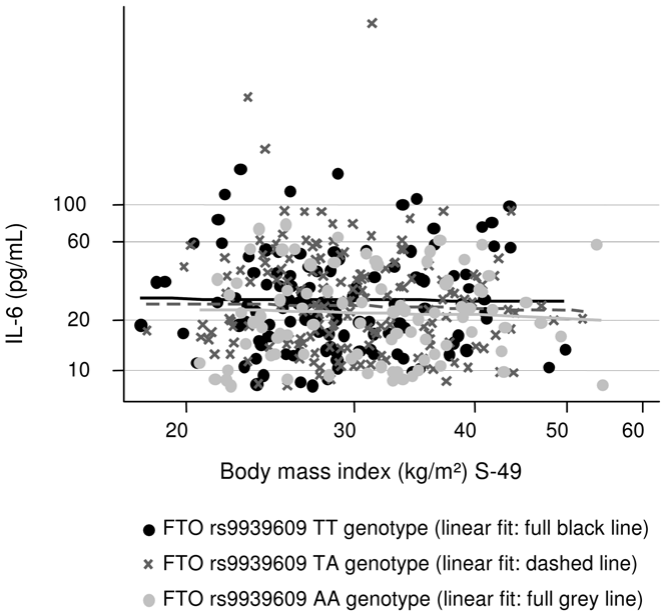
The association between BMI and IL-6 according to *FTO* rs9939609 genotype. *FTO* rs9939609 genotype had no main effect on hs-CRP neither in the crude analysis nor when BMI was taken into account (see Table 3); further, this figure shows there was no interaction between BMI and *FTO* rs9939609 genotype in relation to IL-6 (p = 0.82).

**Figure 3:**
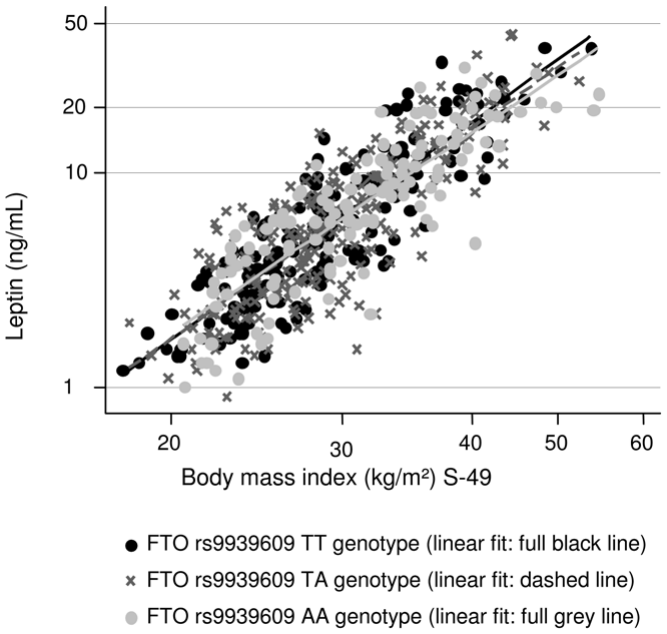
The association between BMI and Leptin according to *FTO* rs9939609 genotype. *FTO* rs9939609 genotype had no main effect on leptin when BMI was taken into account (see Table 3); further, this figure shows there was no interaction between BMI and *FTO* rs9939609 genotype in relation to leptin (p = 0.31).

The results for the linear regressions of hs-CRP, IL-6 and leptin on *FTO* genotype and the anthropometric obesity measures, respectively, are presented in Table 3. *FTO* genotype and hs-CRP were positively associated, e.g. the hs-CRP level increased by 17% per additional A-allele (p = 0.05). However, this association disappeared when BMI and the other obesity markers were taken into account. Similarly, the leptin level increased by 10% (p = 0.04) per additional A-allele, but BMI, WHR and waist circumference accounted for this association. Further, positive associations were seen between BMI, WHR and waist (for given BMI) and hs-CRP and leptin, respectively, and these remained significant when *FTO* genotype was taken into account. As an example, hs-CRP level increased by 20% per 10% increase in BMI (p<0.001), and by 19% (p<0.001) when *FTO* was adjusted for. There were no association between IL-6 and *FTO* genotype and the anthropometric obesity measures, respectively. Furthermore, no interactions between BMI and *FTO* genotype in regard to hs-CRP, IL-6 and leptin, respectively, were observed (Figures 1, 2, 3).

**Table 3:** Effect estimates* with 95% CIs based on regression analyses of the inflammatory markers per the unit of the covariates.

		CRP			IL-6			Leptin		
Model		Estimate*	95% CI	P-value	Estimate*	95% CI	P-value	Estimate*	95% CI	P-value
1	*FTO* rs9939609 (additive)	1.17	1.00; 1.37	0.05	0.91	0.83; 1.01	0.07	1.10	1.00; 1.21	0.04
2	BMI (per 10% increase)	1.20	1.14; 1.26	<0.001	0.99	0.96; 1.02	0.43	1.37	1.35; 1.39	<0.001
3	WHR (per 10% increase)	1.62	1.42; 1.86	<0.001	0.96	0.88; 1.05	0.38	2.05	1.93; 2.18	<0.001
4	Waist (per 10 cm increase)	1.28	1.01; 1.61	0.04	1.00	0.86; 1.17	0.95	1.35	1.26; 1.44	<0.001
	BMI (per 10% increase)	1.00	0.84; 1.19	0.99	0.98	0.88; 1.10	0.78	1.10	1.04; 1.16	<0.001
5	*FTO* rs9939609 (additive)	1.10	0.94; 1.27	0.23	0.92	0.83; 1.01	0.08	0.98	0.93; 1.03	0.42
	BMI (per 10% increase)	1.19	1.14; 1.25	<0.001	0.99	0.96; 1.02	0.57	1.37	1.35; 1.39	<0.001
6	*FTO* rs9939609 (additive)	1.12	0.97; 1.95	0.12	0.91	0.83; 1.01	0.07	1.04	0.97; 1.11	0.27
	WHR (per 10% increase)	1.61	1.41; 1.85	<0.001	0.97	0.88; 1.06	0.44	2.04	1.92; 2.17	<0.001
7	*FTO* rs9939609 (additive)	1.10	0.94; 1.27	0.23	0.92	0.83; 1.01	0.07	0.98	0.94; 1.03	0.43
	Waist (per 10 cm increase)	1.28	1.01; 1.61	0.04	1.01	0.87; 1.17	0.93	1.35	1.26; 1.44	<0.001
	BMI (per 10% increase)	1.00	0.84; 1.19	0.99	0.99	0.88; 1.10	0.80	1.10	1.04; 1.16	<0.001
8	*FTO* rs9939609 (additive)	1.10	0.95; 1.28	0.21	0.91	0.83; 1.01	0.07	0.99	0.94; 1.03	0.56
	BMI (per 10% increase)	1.12	1.04; 1.20	0.003	1.00	0.95; 1.05	0.95	1.32	1.29; 1.35	<0.001
	WHR (per 10% increase)	1.28	1.05; 1.57	0.02	0.97	0.85; 1.10	0.63	1.15	1.08; 1.23	<0.001

Abbreviations: BMI: Body mass index. WHR: Waist-to-hip ratio.

All models were adjusted for age (per year).

*Factor of the change in the inflammatory marker relative to the measurement unit in the predicting covariate (e.g. an estimate of 1.17 for *FTO* genotype in relation to hs-CRP is to be interpreted as a 17% higher hs-CRP-level per additional A-allele).

The analyses were repeated with fat-BMI instead of BMI; however, this did not change the associations. Further adjustment for smoking habits, alcohol consumption, and physical activity also did not change the associations. Regarding hs-CRP, additional analyses were performed based on exclusion of individuals with hs-CRP values above 10 mg/l (31 randomly selected and 34 obese men), leaving all associations fairly unchanged.

Finally, virtually the same pattern of results was obtained by logistic regression analyses of the dichotomized values of the inflammatory markers (Table S2).

## Discussion

In the present study of middle-aged Danish men positive associations between hs-CRP, leptin and BMI were observed. The associations did not differ across *FTO* rs9939609 genotype. For IL-6, there was a non-significant tendency to lower levels by the *FTO* A-allele, i.e. opposite to the expectation, independent of the fatness variables. There were no associations between IL-1β, IL-10, IL-18, mip1α, mip1β, sTNFα-R1, TGF-β, and TNF-α and *FTO* genotype or BMI, respectively, in this cohort. Generally, the confidence intervals were so narrow that it seems fair to suggest that all together the *FTO* rs9939609 A-allele does not have important fatness-independent effects on systemic inflammatory markers or adipokines.

A fatness-independent association between the *FTO* rs9939609 A-allele and hs-CRP was observed in a recent German study [12], and the effect was persistent even when prevalent myocardial infarction, stroke and diabetes were taken into account. The participants in the German study were sampled among the general population, with a mean body mass index (BMI) of 26.8 kg/m^2^ among men and 25.7 kg/m^2^ among women. We did not find an association between *FTO* rs9939609 and systemic inflammatory markers among generally healthy middle-aged men representing a broad range of BMI. The restriction in the inclusion of men to those without known diseases or regular medication may explain why we did not find an association between BMI and a range of adipokines generally known to associate with increased BMI [6]. Whether it also influenced our findings in relation to hs-CRP and *FTO* rs9939609 is only speculative. Regarding the circulating hormone leptin, a study on diabetics of both sexes found that *FTO* rs9939609 affected circulating leptin levels, yet the effect was accounted for by BMI [24]. In our data of relatively healthy subjects the *FTO* rs9939609 A-allele also did not have fatness-independent effects on the circulating levels of leptin. Thus, there is no reason to believe that the *FTO* rs9939609 A-allele has any functional impact on leptin in addition to the effect on fatness. Finally, we were surprised that the *FTO* A-allele tended to lower IL-6 levels, since we would have expected obesity to lead to increased IL-6 levels [3].

We have previously shown that *FTO* rs9939609 had a fatness-independent effect on mortality and morbidity when analysed in a dominant model. We found that carriers of the A-allele had nearly twice the mortality compared to non-carriers irrespective of BMI [25]. In relation to vascular diseases, we observed a tendency towards an increased risk among A-allele carriers illustrated by a hazard ratio of prevalent vascular disease at time of death of 1.43 (95% CI, 0.87–2.37; p = 0.15) vs. non-carriers [25]. A Scottish study of nearly 5000 diabetics of both sexes found that A-allele carriers of the *FTO* rs9939609 had more than twice the risk of a myocardial infarction or cardiovascular death compared with non-carriers, when adjusted for age, gender, BMI, smoking and history of myocardial infarction [26]. Human adipose tissue is heterogeneous in its metabolic activity, which lead the authors of the German study to suggest that the sites in the adipose tissue that are more sensitive to infiltration of immune cells might be expanded preferentially among A-allele carriers, resulting in a increased risk of cardiovascular disorder [12]. However, our results do not support the hypothesis that the increased morbidity and mortality could be explained by a fatness-independent effect of the *FTO* rs9939609 A-allele on systemic inflammation.

There are several strengths of this study, but also limitations that should to be taken into consideration. The study is based on a unique, well-defined large background study population of white men with the obese and random sample derived from the same genetically quite homogenous population, hence eliminating population stratification. The study design and size of the study population implied that the confidence limits on the non-significant associations were fairly narrow, meaning that presence of any true major associations related to the *FTO* rs9939609 genotype are very unlikely. A limitation of our study relates to the fact that the biology of the *FTO* rs9939609 is not fully known. Further, the investigated SNP is located in the first intron of the gene, which according to the current knowledge may not be biologically functioning, so even though only speculative, there is a possibility that this SNP is in linkage disequilibrium with a functional variant within the *FTO* or nearby genetic regions [19]. Moreover, the inflammatory markers were measured only once, which implies that the levels were subject to random variation due to fluctuations over time. Repetitive measurements would potentially provide a more stable mean measure for the single individual. However, random variation would only introduce bias if this variation were dependent on genotype, which we do not suspect.

In summary, we found positive associations between hs-CRP, leptin and BMI; however, the associations did not differ across *FTO* rs9939609 genotype. There were no associations between IL-1β, IL-6, IL-10, IL-18, mip1α, mip1β, sTNFα-R1, TGF-β, and TNF-α or *FTO* genotype and BMI, respectively, in this cohort. All together our results do not support the tentative evidence that the *FTO* rs9939609 A-allele have fatness-independent effects on systemic inflammation.

## Supporting Information

Table S1 **Geometric mean (95% confidence intervals) of the inflammatory markers according to** ***FTO*** **rs9939609 genotype for the two cohorts.**
(DOC)

Table S2 Odds ratios* with 95% CIs of the inflammatory markers per additional A-allele of *FTO* rs9939609 adjusted for age and body mass index.
(DOC)
