# Supplementary material for: Influences of the Common FTO rs9939609 Variant on Inflammatory Markers Throughout a Broad Range of Body Mass Index
Source: PLoS One. 2011 Jan 5;6(1):e15958. doi: 10.1371/journal.pone.0015958 (PMC3016333; doi:10.1371/journal.pone.0015958)
Supplement: Table S1 — Geometric mean (95% confidence intervals) of the inflammatory markers according to FTO rs9939609 genotype for the two cohorts. (DOC) [file pone.0015958.s001.doc]

| Inflammatory marker | Cohort | N | TT |  | **TA** |  | **AA** |  | **P*** | **P^†^** |
| --- | --- | --- | --- | --- | --- | --- | --- | --- | --- | --- |
|  |  |  | Mean | 95% CI | Mean | 95% CI | Mean | 95% CI |  |  |
| hs-CRP (mg/l) | Random sample | 305 | 0.90 | 0.69; 1.17 | 1.00 | 0.82; 1.23 | 1.42 | 0.98; 2.08 | 0.05 |  |
|  | Obese sample | 224 | 1.49 | 1.07; 2.07 | 1.80 | 1.38; 2.35 | 1.51 | 1.09; 2.11 | 0.96 | 0.22 |
| IL-1β (pg/ml) | Random sample | 59 | 16.28 | 11.35; 23.35 | 15.36 | 10.55; 22.35 | 13.22 | 9.52; 18.37 | 0.58 |  |
|  | Obese sample | 45 | 16.61 | 12.21; 22.59 | 17.09 | 10.75; 27.18 | 12.08 | 9.78; 14.93 | 0.43 | 0.76 |
| IL-6 (pg/ml) | Random sample | 210 | 24.95 | 21.34; 29.18 | 27.69 | 23.63; 32.45 | 22.42 | 18.18; 27.65 | 0.64 |  |
|  | Obese sample | 167 | 27.52 | 22.22; 34.09 | 24.22 | 20.84; 28.16 | 20.40 | 17.23; 24.16 | 0.03 | 0.22 |
| IL-10 (pg/ml) | Random sample | 84 | 23.41 | 15.87; 34.52 | 23.10 | 17.33; 30.79 | 24.30 | 14.93; 39.55 | 0.94 |  |
|  | Obese sample | 61 | 17.81 | 14.89; 21.30 | 22.75 | 15.88; 32.61 | 17.85 | 12.04; 26.47 | 0.93 | 0.94 |
| IL-18 (pg/ml) | Random sample | 297 | 337.91 | 293.71; 388.77 | 336.13 | 296.72; 380.79 | 384.64 | 314.30; 470.72 | 0.37 |  |
|  | Obese sample | 222 | 354.21 | 291.75; 430.04 | 385.35 | 331.29; 448.22 | 363.22 | 305.38; 432.02 | 0.86 | 0.64 |
| TNF-α (pg/ml) | Random sample | 105 | 16.81 | 12.77; 22.12 | 18.78 | 13.91; 25.35 | 15.49 | 11.98; 20.02 | 0.91 |  |
|  | Obese sample | 92 | 16.79 | 12.72; 22.15 | 15.39 | 12.17; 19.47 | 12.96 | 11.18; 15.03 | 0.17 | 0.49 |
| STNFα-R1 (pg/ml) | Random sample | 254 | 564.79 | 473.43-673.78 | 605.37 | 514.42; 712.41 | 525.34 | 427.41; 645.70 | 0.81 |  |
|  | Obese sample | 192 | 604.76 | 488.37; 748.89 | 657.56 | 561.90; 769.52 | 602.50 | 502.59; 722.26 | 0.95 | 0.91 |
| TGF-β (pg/ml) | Random sample | 221 | 143.07 | 125.60; 162.97 | 150.79 | 137.64; 165.20 | 137.74 | 125.29; 151.43 | 0.83 |  |
|  | Obese sample | 150 | 144.99 | 128.13; 164.08 | 151.08 | 137.55; 165.95 | 149.04 | 134.60; 165.03 | 0.74 | 0.70 |
| MiP-1α (pg/ml) | Random sample | 208 | 41.98 | 36.27; 48.59 | 39.67 | 35.27; 44.63 | 51.46 | 37.23; 71.21 | 0.29 |  |
|  | Obese sample | 142 | 44.71 | 36.20; 55.21 | 38.82 | 32.85; 45.87 | 43.02 | 32.55; 56.87 | 0.82 | 0.39 |
| MiP1β (pg/ml) | Random sample | 309 | 138.13 | 121.89; 156.53 | 139.89 | 125.46; 155.98 | 144.04 | 120.56; 172.08 | 0.71 |  |
|  | Obese sample | 228 | 140.87 | 119.51; 166.06 | 145.61 | 129.16; 164.15 | 142.37 | 123.17; 164.56 | 0.93 | 0.81 |
| Leptin (ng/ml) | Random sample | 319 | 3.88 | 3.43; 4.39 | 3.92 | 3.55; 4.33 | 4.03 | 3.33; 4.87 | 0.74 |  |
|  | Obese sample | 229 | 10.64 | 8.87; 12.76 | 9.83 | 8.53; 11.34 | 10.55 | 9.11; 12.21 | 0.96 | 0.86 |

*Test for trend

†P for interaction between the randomly selected and obese cohort and *FTO* genotype
