# Supplementary material for: Influences of the Common FTO rs9939609 Variant on Inflammatory Markers Throughout a Broad Range of Body Mass Index
Source: PLoS One. 2011 Jan 5;6(1):e15958. doi: 10.1371/journal.pone.0015958 (PMC3016333; doi:10.1371/journal.pone.0015958)
Supplement: Table S2 — Odds ratios* with 95% CIs of the inflammatory markers per additional A-allele of FTO rs9939609 adjusted for age and body mass index. (DOC) [file pone.0015958.s002.doc]

|  | N of detectable measures† | | |  |  |  |  |
| --- | --- | --- | --- | --- | --- | --- | --- |
| Inflammatory marker | Below | Between | Above | Median‡ | **OR** | **95% CI** | **P-value** |
| hs-CRP (mg/l) | 14 | 529 | 0 | 1.10 | 1.10 | 0.93; 1.54 | 0.16 |
| IL-1β (pg/ml) | 437 | 104 | 1 | 8.00 | 0.90 | 0.67; 1.21 | 0.16 |
| IL-6 (pg/ml) | 164 | 377 | 1 | 15.83 | 0.95 | 0.75; 1.20 | 0.65 |
| IL-10 (pg/ml) | 395 | 145 | 2 | 8.00 | 0.98 | 0.75; 1.27 | 0.86 |
| IL-18 (pg/ml) | 21 | 519 | 2 | 366.27 | 1.13 | 0.89; 1.43 | 0.31 |
| TNF-α (pg/ml) | 344 | 197 | 1 | 8.00 | 1.04 | 0.81; 1.32 | 0.82 |
| STNFα-R1 (pg/ml) | 95 | 450 | 1 | 439.65 | 1.03 | 0.81; 1.30 | 0.82 |
| TGF-β (pg/ml) | 171 | 425 | 0 | 111.84 | 1.11 | 0.88; 1.40 | 0.40 |
| MiP-1α (pg/ml) | 191 | 350 | 1 | 25.14 | 0.96 | 0.76; 1.21 | 0.74 |
| MiP1β (pg/ml) | 5 | 537 | 0 | 138.82 | 0.90 | 0.71; 1.14 | 0.39 |
| Leptin (ng/ml) | 0 | 548 | 0 | 5.70 | 1.29 | 0.92; 1.82 | 0.15 |

Abbreviations: OR: odds ratio

*The odd ratios were estimated by a logistic regression model with the dichotomized values of all measurements of the inflammatory markers as response variable and the *FTO* rs9939609 genotype, BMI and age as covariates

†Number of measures below, between and above the detectable concentration limits. The minimum detectable concentration was 0.005 mg/l for CRP, 8 pg/ml for IL-1β, IL-6, IL-10 and TNF-α; 20 pg/ml for IL-18, MIP-α and MIP-β; 80 pg/ml for TGF-β; 156 pg/ml for sTNFα-R1; 0.5 ng/ml for leptin. The upper measurable concentration was 4,000 pg/ml for IL-1β, Il-6, IL-10 and TNF-α; 10,000 pg/ml for IL-18, MIP-α and MIP- β; 40,000 for TGF-β pg/ml; 80,000 pg/ml for sTNFα-R1.

‡Median values used for the dichotomization of the inflammatory markers
